# The Colorado State Dental Association

**Published:** 1892-11

**Authors:** Florance D. Covert

**Affiliations:** 306 Mack Blk., Denver, Colo.


### The Colorado State Dental Association.

The sixth annual session of the Colorado State Dental Association was held at Denver, Colorado, June 7 to 9, 1892. The following papers were read: "Dental Caries," Dr. P. T. Smith, Denver; "A Few words about Gold," Dr. A. H. Sawins, Denver; "Anæsthetics," Dr. T. C. Chamberlain, Colorado Springs; "Dental Education," Dr. Donaldson, Denver.

A very interesting clinic on Removable Facings in Bridges was given by Dr. Bryant, Denver. A fund of $80.00 was raised by private subscription to assist the board of Dental Examiners in prosecuting violators of the dental law, $50.00 of which was appropriated for the immediate use of the board. All dentists were urged to join the Dental Protective Association. Six new members were elected, viz.: Drs. Lloyd S. Gilbert, Denver, Colo.; Robt. Ketlner, Trinidad, Colo.; Issac Bryant, La Junta, Colo.; Lewis M. Raub, Central City, Colo.; G. B. Harlan, Montrose, Colo., A. E. Baker, Idaho Springs, Colo.

Appropriate resolutions were passed upon the death of Dr. R. J. Burns, of Denver. The resignation of W. K. Dameron, Denver, was accepted.

Liability of dentists for jury duty was fully discussed, and it being developed that no law existed exempting dentists, a committee consisting of Drs. R. B. Weiser, J. M. Norman and S. Davis, was appointed to secure the passage of such a law and to look after all legislation affecting dentists.

The following officers were elected for the ensuing year: President, Dr. J. M. Norman, Denver; First Vice-President, Dr. J. H. Beals, Denver; Second Vice-President, Dr. Rob't Keltner, Trinidad; Recording Secretary, Dr. W. A. Smith, Salida; Corresponding Secretary, Dr. Sarah May Townsand, Denver; Treasurer, Dr. Wm. Smedley, Denver.

The meeting adjourned to meet on the first Tuesday in June, 1893, at Denver, Colo.

Florance D. Covert, Cor. Secretary,
306 Mack Blk., Denver, Colo.

